# From Transfusion Medicine to Vaccines: The Enduring Impact of Karl Landsteiner

**DOI:** 10.7759/cureus.67930

**Published:** 2024-08-27

**Authors:** Mayank Sharma, Sonali G Choudhari, Pankaj C Jambholkar

**Affiliations:** 1 Community Medicine, Jawaharlal Nehru Medical College, Datta Meghe Institute of Higher Education and Research, Wardha, IND

**Keywords:** public health, noble prize, transfusion medicine, immunology, abo blood group, historical vignette

## Abstract

This is the biography of the Nobel Prize winner Karl Landsteiner who divided human blood into groups according to the presence of naturally occurring agglutinating antibodies. His research eventually led to the establishment of safe transfusion practices. Before his discovery, transfusions of blood were given to patients in need from animals like sheep or randomly chosen human donors, often with disastrous results. Millions of lives were genuinely saved by Landsteiner's discovery. He established the foundation for the creation of the polio vaccine by determining that a microbe causes poliomyelitis. Additionally, Landsteiner contributed to the identification of the syphilis-causing microbes. This biography is a tribute to the legend Karl Landsteiner.

## Introduction and background

**Figure 1:**
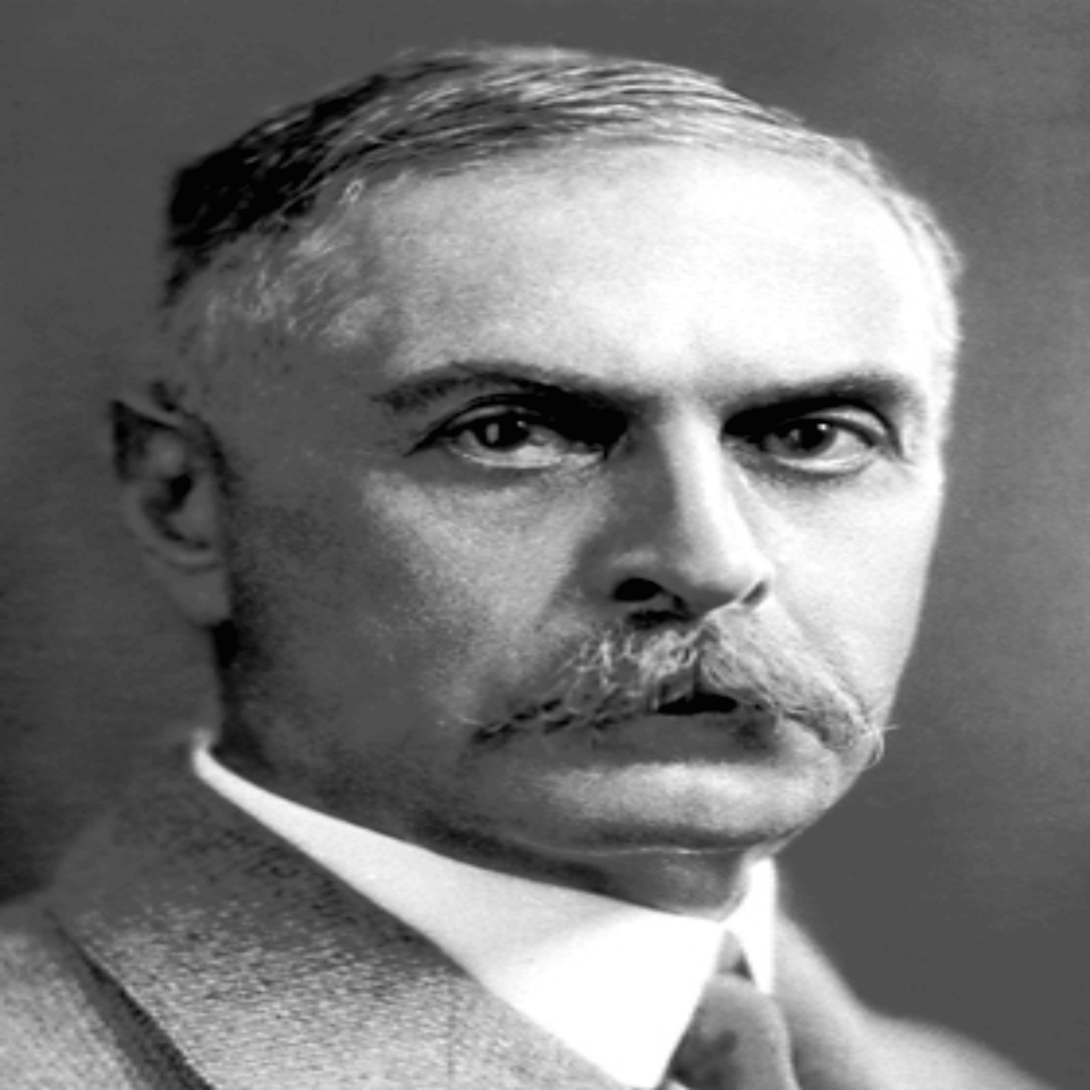
Karl Landsteiner (June 14, 1868 - June 26, 1943) Image courtesy of Wikimedia Commons (public domain).

Karl Landsteiner (Figure 1) was an Austrian-American immunologist and pathologist who was awarded the Nobel Prize in Physiology or Medicine in 1930 for his work developing the ABO blood typing system, which has led to the widespread use of blood transfusion in medicine. He also discovered the major blood groups. At the University of Vienna, where he started medical school, he became interested in chemistry. He took a year off to finish his military duty, then went back to school to complete his degree. At the age of 23, he earned his M.D. in 1891 [1].

After that, he worked in the labs of the greatest chemists of all time, with whom he published numerous papers and became interested in immunology. Between 1897 and 1920, he worked as an assistant and then an associate professor in Vienna, where he conducted research on poliomyelitis, proving it was contagious and identifying its virus. He started testing the sera and red blood cells of the scientists who worked in his laboratory in 1900. He found that the blood of some scientists caused the blood of others to clump together, indicating the possibility of at least two blood groups. In a paper that he published, he first mentioned the agglutination of human blood and suggested that, rather than having a pathological cause, this was related to the individuality of each person's blood. The four blood groups were identified as a result of this important conclusion [2].

The Imperial Society of Physicians in Vienna (1902), the French Legion of Honor (1911), the Hans Aronson Foundation Prize (1926), the German Academy of Sciences (1927), the Paul Ehrlich Medal (1930), the Nobel Laureate in Physiology or Medicine (1930), the Member of the National Academy of Sciences (1932), the Dutch Red Cross Medal (1933), the American Philosophical Society (1935), the Honorary Foreign Member of the Royal Society (1941), and the Albert Lasker Clinical Medical Research Award (1946) are just a few of the numerous honors and awards that bear his name [3].

## Review

Early life and career

Karl Landsteiner was born on June 14, 1868, in Vienna, Austria. He was only six years old when his father, Leopold Landsteiner, the editor-in-chief of Die Presse and a well-known Viennese journalist, passed away at the age of 56. As a result, he and his mother Fanny, née Hess, had a close relationship from 1837 to 1908. He enrolled in the University of Vienna to pursue his medical studies after passing the Matura exam from a secondary school in Vienna, where he also completed his doctoral thesis in 1891. He wrote and published an essay about how diets affect blood composition while he was still a student. He studied chemistry under Hermann Emil Fischer in Würzburg, Eugen Bamberger in München, and Arthur Rudolf Hantzsch in Zürich between 1891 and 1893. During that time, he published several works, some of them in collaboration with his instructors [4].

He developed a fascination with organic chemistry while pursuing his medical degree. He chose to work in research instead of becoming a practicing physician. He worked for several years with some of the greatest names in organic chemistry, such as Emil Fischer, in Germany and Switzerland, where he learned cutting-edge laboratory techniques. He continued his medical education by working in the surgical clinic at the University of Vienna [2].

Multifaceted medical pioneer and haematologist

Blood studies were only one aspect of Landsteiner’s research focus. He is also responsible for providing important insights regarding the poliomyelitis syndrome. He recognized, in collaboration with the Austrian physician Erwin Popper, that an infection is the root cause of poliomyelitis. He could not find a conducive environment for his research in Austria following World War I, so in 1919 he left the country and moved to Den Haag, then to the USA, where he worked at the Rockefeller Institute for Medical Research. There, in 1940, he and Philip Levine and Alexander Solomon Wiener discovered the Rhesus factor [5].

Role in infectious diseases

Landsteiner and Erwin Popper discovered a virus, which they later named the poliovirus, to be the cause of poliomyelitis a little more than a century ago. This important discovery simultaneously served as the foundation for the precautions taken today to stop the terrible poliovirus epidemics from breaking out. The World Health Organization began its eradication program to eradicate the virus from the earth in 1988. The symposium addressed the current understanding of poliovirus biology while commemorating the discovery of the virus. Prospects for the eradication program were assessed, with a focus on the reasons why some nations have not yet been able to stop the spread of the poliovirus in its wild form. The role of inactivated poliovirus vaccines in the eradication program and the upkeep of a world free of polioviruses, whenever this objective should be accomplished, were also topics of discussion [6].

Discovery of blood groups and challenges 

Human blood was one of the subjects he studied and thought to be fascinating. Before the discovery of ABO blood grouping, he faced several challenges. Firstly, he often had to conduct research without adequate funding, which constrained his ability to access the latest equipment and materials. Secondly, the concept of blood groups was revolutionary and initially met with skepticism. The medical community was resistant to the idea that blood types could determine the success of transfusions and lastly, At the time of his research, immunology was not well understood. The mechanisms behind immune reactions were poorly defined, making his work even more challenging. Although transfusions, or giving someone another person's blood, had been attempted, the success of these procedures was essentially luck-based. In certain cases, the patient made a full recovery; in other cases, the patient experienced a catastrophic event. Individuals frequently bled to death from ulcers, accidents, and difficult childbirths before he learned that people had different blood group types. Nobody realized that blood can have four different types: A, B, AB, and O. Blood donors may react negatively to recipients if they receive the incorrect type of blood, which could result in death. When the incorrect type of blood is given to someone, a horrible reaction starts. The red blood cells may react with the new blood cells as a result, which could lead to a cascade of other issues, including the cells lysing, or breaking down, in the blood vessels. Hemoglobin that can harm the kidneys and cause death is released as a result. Landsteiner found in 1901 that blood from different people had different characteristics that rendered it "incompatible" with blood from other people who did not share those characteristics. The blood types A, B, and O were found by him. Blood transfusions became a common practice thanks to his discovery of the differences and identification of the groups that shared similarities. This set the path for numerous other medical practices, like surgery, blood banks, and transplants, that we take for granted today. Blood transfusions can now be done safely thanks to Landsteiner's work on blood typing. His research was published in 1901 in Wiener Klinische Wochenschrift, the Central European Journal of Medicine. For saving lives, he was awarded the Nobel Prize in Physiology or Medicine in 1930 [7].

Discovery of the Rh factor

Further blood grouping discoveries were made by Landsteiner in 1923 while he was employed at the Rockefeller Institute for Medical Research in New York. Blood groups M, N, and P which were initially employed in paternity testing were identified with his assistance. Due to studies done on rhesus monkeys, Landsteiner and Alexander Wiener identified the Rh factor blood group in 1940. Blood cells that have the Rh factor on them are Rh-positive (Rh+). An Rh-negative (Rh-) type is indicated by the lack of the Rh factor. This finding made it possible to match Rh blood types in order to avoid incompatibility reactions when receiving transfusions [8].

Syphilis

The next significant discovery made by him was related to the sexually transmitted illness syphilis. He was the first scientist to find a way to infect monkeys with syphilis in 1905, which allowed for more investigation. The following year, he discovered, in collaboration with Viktor Mucha, that syphilis could be diagnosed by using dark-field microscopy to identify the Treponema pallidum bacteria that causes the illness [9].

Father of hematology and immunology

His contributions to the fields of immunology and hematology have made him well-known. Although he initially struggled to manage the funding for his research, he persisted. In the end, he made some of the most significant discoveries in biology. His work in isolating dead viruses led to the discovery of vaccines against a few deadly diseases. As a result, his medical research is responsible for the lives of many people. He had the option to practice medicine after completing his studies, but he decided to focus on medical research instead. He has made enormous contributions to the fields of hematology and immunology [10].

Social challenges and legacy

His career spanned periods of significant disturbance, including World War I and the interwar years, which affected funding, collaboration opportunities, and the stability necessary for sustained research. As a person of Jewish descent, he faced social prejudice and discrimination, which influenced his decision to emigrate from Austria to the United States in search of better opportunities and a safer environment for his work. The aftermath of global conflicts led to economic instability, impacting research funding and resource availability, forcing scientists like him to work under constrained conditions. Despite these social challenges, his resilience and innovative spirit led to monumental contributions to medicine, including the discovery of the ABO blood group system, which has saved countless lives and laid the groundwork for modern transfusion medicine and immunology. He made significant contributions to hematology, but his work also had a significant impact on virology. His discovery that a microbe causes poliomyelitis, along with Constantin Levaditi's, set the stage for the creation of the polio vaccine. In terms of public health and the prevention of infectious diseases, this was a huge advancement. In 1930, he received the Nobel Prize in Physiology or Medicine in recognition of his outstanding achievements. His work has impacted legal and forensic sciences as well as medicine because blood typing is used in police investigations and paternity tests [11,12].

## Conclusions

Karl Landsteiner was a visionary scientist whose discoveries have had a lasting impact on medicine and human health. His identification of the ABO blood group system and the Rh factor revolutionized transfusion medicine, making it possible to safely and effectively transfer blood between individuals. His contributions to immunology, particularly his work on antigens and antibodies, have provided the foundation for countless medical advancements, from vaccines to therapeutic antibodies. His contributions continue to resonate, ensuring that his name will be remembered as one of the greatest scientific minds of the 20th century.
